# Chordae tendineae rupture: a rare cause of tricuspid regurgitation following coronary artery bypass grafting

**DOI:** 10.1186/s12871-025-03270-0

**Published:** 2025-07-29

**Authors:** Craig Stewart, Dana Wu, Jong Moo Steve Kim, Jian Ye, Julena Foglia

**Affiliations:** 1 Department of Anesthesiology & Perioperative Care, Vancouver General Hospital, JPPN 2nd Floor, Room 2449, 899 West 12th Ave, Vancouver, BC V5Z 1M9 Canada; 2 Department of Anesthesiology, Pharmacology, and Therapeutics, University of British Columbia, 217–2176 Health Sciences Mall, Vancouver, BC V6T 1Z3 Canada; 3 Faculty of Medicine, University of British Columbia, 317 - 2194 Health Sciences Mall, Vancouver, BC V6T 1Z3 Canada; 4 Division of Cardiovascular Surgery, University of British Columbia, Vancouver, BC Canada

**Keywords:** Transesophageal echocardiography, Cardiac surgical procedures, Tricuspid regurgitation anesthesiology, Coronary artery bypass grafting, Papillary muscle, Chordae tendineae

## Abstract

**Background:**

Tricuspid regurgitation is classified broadly by its etiology being either structural or functional. New or worsening tricuspid regurgitation upon weaning from cardiopulmonary bypass is rare and often functional. However, iatrogenic structural causes are possible and should be investigated with a high index of suspicion when issues separating from cardiopulmonary bypass arise. Currently, isolated coronary artery bypass grafting is not a class I indication for intraoperative transesophageal echocardiography, leading to significant practice variation. However, intraoperative transesophageal echocardiography, even during routine isolated coronary artery bypass grafting, can be a valuable tool in differentiating between functional and structural causes of post-cardiopulmonary bypass tricuspid regurgitation, allowing for prompt surgical intervention when required.

**Case presentation:**

We report a case of a 63-year-old female, American Society of Anesthesiologists physical status 4, who underwent an isolated coronary artery bypass grafting surgery and subsequently developed difficulty weaning from cardiopulmonary bypass after completion of revascularization. Intraoperative transesophageal echocardiography revealed severe tricuspid regurgitation with evidence of right ventricular dysfunction which was initially presumed to be functional in etiology. However, further investigation with transesophageal echocardiography revealed a flail posterior tricuspid valve leaflet with an anteriorly directed jet. The decision was made to re-institute cardiopulmonary bypass for prompt surgical correction of what was determined to be an acutely ruptured chordae tendineae originating from an anomalous papillary muscle of the posterior tricuspid leaflet. Successful surgical repair was performed with neochord implantation and a 26 mm tricuspid annuloplasty ring. Final intraoperative transesophageal echocardiography demonstrated mild residual tricuspid regurgitation with normal biventricular size and systolic function and the patient was subsequently weaned off cardiopulmonary bypass without issue.

**Conclusions:**

We describe a case of a ruptured chordae tendineae causing new severe tricuspid regurgitation after an isolated coronary bypass grafting surgery. The use of intraoperative transesophageal echocardiography was essential for the prompt diagnosis and surgical correction of this rare structural cause of post-cardiopulmonary tricuspid regurgitation. This case lends support to the routine use of intraoperative transesophageal echocardiography in isolated coronary artery bypass grafting surgery.

**Supplementary Information:**

The online version contains supplementary material available at 10.1186/s12871-025-03270-0.

## Background

Tricuspid regurgitation (TR) can be classified broadly into structural or functional etiologies. In patients undergoing Coronary Artery Bypass Grafting (CABG), functional TR can be related to pathological remodeling of the right heart due to coronary artery disease (CAD) or ischemic cardiomyopathy [1]. Worsening TR post cardio-pulmonary bypass may be related to changes in volume status, myocardial dysfunction from poor myocardial protection, ventricular pacing, and/or acute increases in right ventricular afterload [2, 3]. Therefore, correction of post-CPB TR is often aimed at addressing these functional causes including optimizing preload, augmenting ventricular function, and restoring a native narrow complex rhythm. Structural causes of post-CPB TR are possible and often more difficult to elucidate intraoperatively.

Currently, isolated CABG surgery alone is not a class I clinical indication for intraoperative TEE monitoring per most recent practice guidelines from the American Society of Echocardiography and the Society of Cardiovascular Anesthesiologists [4]. Thus, the use of intraoperative TEE in this surgical cohort is variable and typically anesthesiologist and surgeon-dependent. However, as evidence for the clinical utility of TEE continues to evolve, so will the need for updated clinical recommendations on its use. This case showcases the utility of intraoperative TEE during routine, isolated CABG for the prompt diagnosis and surgical management of post-CPB TR from a rare and iatrogenic structural cause.

## Case presentation

We report a 63-year-old female, American Society of Anesthesiologists physical status 4, with a past medical history significant for hypertension medically-managed and dyslipidemia, admitted with a diagnosis of unstable angina requiring inpatient surgical revascularization. She presented with a 2-month history of worsening exertional chest pain in the context of a recent coronary computed tomography angiography (CCTA) which showed severe CAD with high-grade stenosis of the mid left anterior descending (LAD) coronary artery, in addition to moderate stenosis of the distal left circumflex (LCx) and mid right coronary artery (RCA). Subsequent inpatient cardiac catheterization revealed diffuse 3-vessel coronary disease with 80% occlusion of the mid LAD, mid LCx, and mid RCA. Preoperative transthoracic echocardiography (TTE) showed a normal left ventricular ejection fraction (LVEF) of 60%, no regional wall motion abnormalities, trivial TR and mitral regurgitation (MR), and normal right ventricular size and function. Cardiac surgery was consulted, and the patient was accepted for inpatient CABG.

On the day of surgery, following written informed consent, the patient was positioned supine on the operating table with standard Canadian Anesthesiologists’ Society monitors applied. Adequate peripheral intravenous access was obtained in addition to a pre-induction right radial arterial line. After sufficient time for preoxygenation, general anesthesia was induced and the airway was secured with a combination of sufentanil, lidocaine, propofol, and rocuronium. General anesthesia was maintained on a combination of sevoflurane and infusions of propofol and sufentanil. A TEE probe was inserted for intraoperative monitoring as was standard practice of the cardiac anesthesiologist conducting the case. Central venous access was obtained with a 16 cm, 7 Fr triple lumen catheter and a 10 cm, 8.5 Fr sheath introducer inserted into the right internal jugular vein under ultrasound guidance. An activated clotting time (ACT) was drawn and resulted at 130 s. Pre-CPB TEE assessment was performed after line placement and revealed preserved biventricular function with trace TR and MR.

**Fig. 1:**
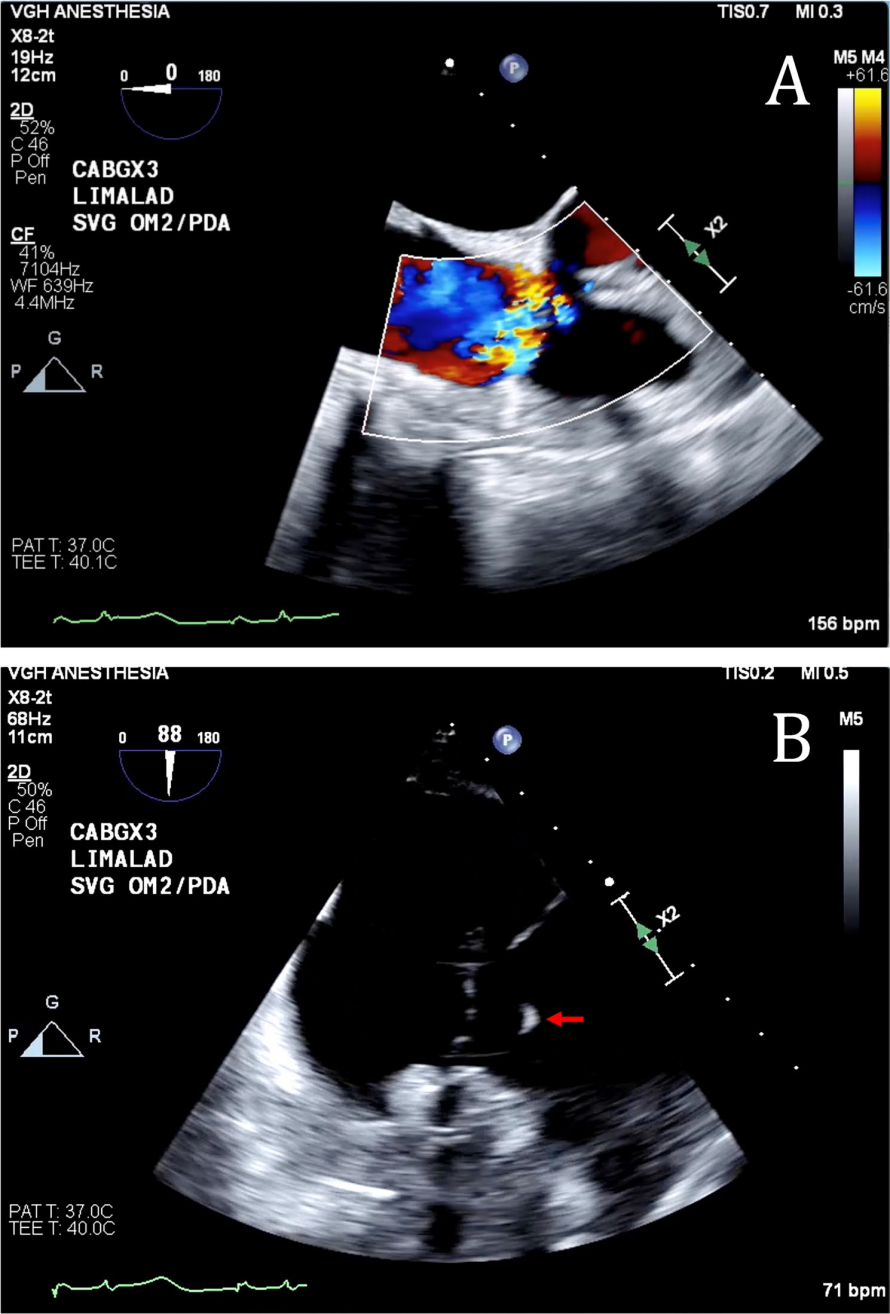
Intraoperative transesophageal echocardiography showing: (**A**) severe tricuspid regurgitation with an anteriorly directed jet on mid esophageal view and (**B**) a flail segment (denoted by the red arrow) of the tricuspid valve on a deep transgastric right ventricular view

Sternotomy was performed uneventfully. The patient was placed on CPB with routine aorta-right atrial cannulation after a loading dose of intravenous unfractionated heparin. Appropriate anticoagulation was confirmed with an ACT. Myocardial protection was performed with antegrade blood cardioplegia via the aortic root as well as the individual saphenous vein grafts. Warm cardioplegia was used for the initial induction dose of 1 L, and after diastolic arrest of the heart, switched to cold. During aortic cross clamping, 200–300 milliliters of cardioplegia was delivered intermittently every 10–15 min as per our institution’s standard cardioplegia protocol. Warm cardioplegia was given prior to removal of the aortic cross clamp. Coronary revascularization was achieved with bypass grafting from the left internal mammary artery (LIMA) to LAD coronary artery and saphenous vein grafts to the posterior descending artery (PDA) and the first obtuse marginal (OM1) coronary artery. Direct flow measurement was checked in each saphenous vein graft after distal anastomosis with a flow of 30 milliliters per minute at a perfusion pressure of 130 mmHg. The duration of the first CPB pump run was 89 min with an aortic cross clamp time of 68 min. Upon weaning from CPB, standard Doppler was used to confirm a biphasic signal in all grafts. However, TEE revealed severe TR with evidence of right ventricular volume overload (Fig. 1, Additional File 1). Left ventricular function was normal with no regional wall motion abnormalities. The severe TR was initially presumed to be functional in etiology. However, further investigation with intraoperative TEE uncovered the appearance of a flail posterior tricuspid valve (TV) leaflet with an anteriorly directed jet (Fig. 1, Additional File 2). A second senior cardiac surgeon with expertise in complex TV repair was consulted intraoperatively. Therefore, the decision was made to re-institute CPB for surgical correction.

Inspection of the TV through an incision made in the right atrium revealed an acutely ruptured posterior leaflet primary chord from its small papillary muscle origin. This ruptured chordae tendineae was seen to originate from an anomalous papillary muscle inserting into the anteroseptal wall of the right ventricle. The TV and chordae looked morphologically normal. The repair was performed with neochord implantation using 5 − 0 Gore-Tex ePTFE sutures (W. L. Gore & Associates) to attach the posterior leaflet edge to the anomalous papillary muscle. Partial wean from CPB showed mild-moderate residual TR. Therefore, the right atrium was opened again and a 26 mm Edwards Physio tricuspid annuloplasty ring (Edwards Lifesciences) was implanted to support the repair.

**Fig. 2:**
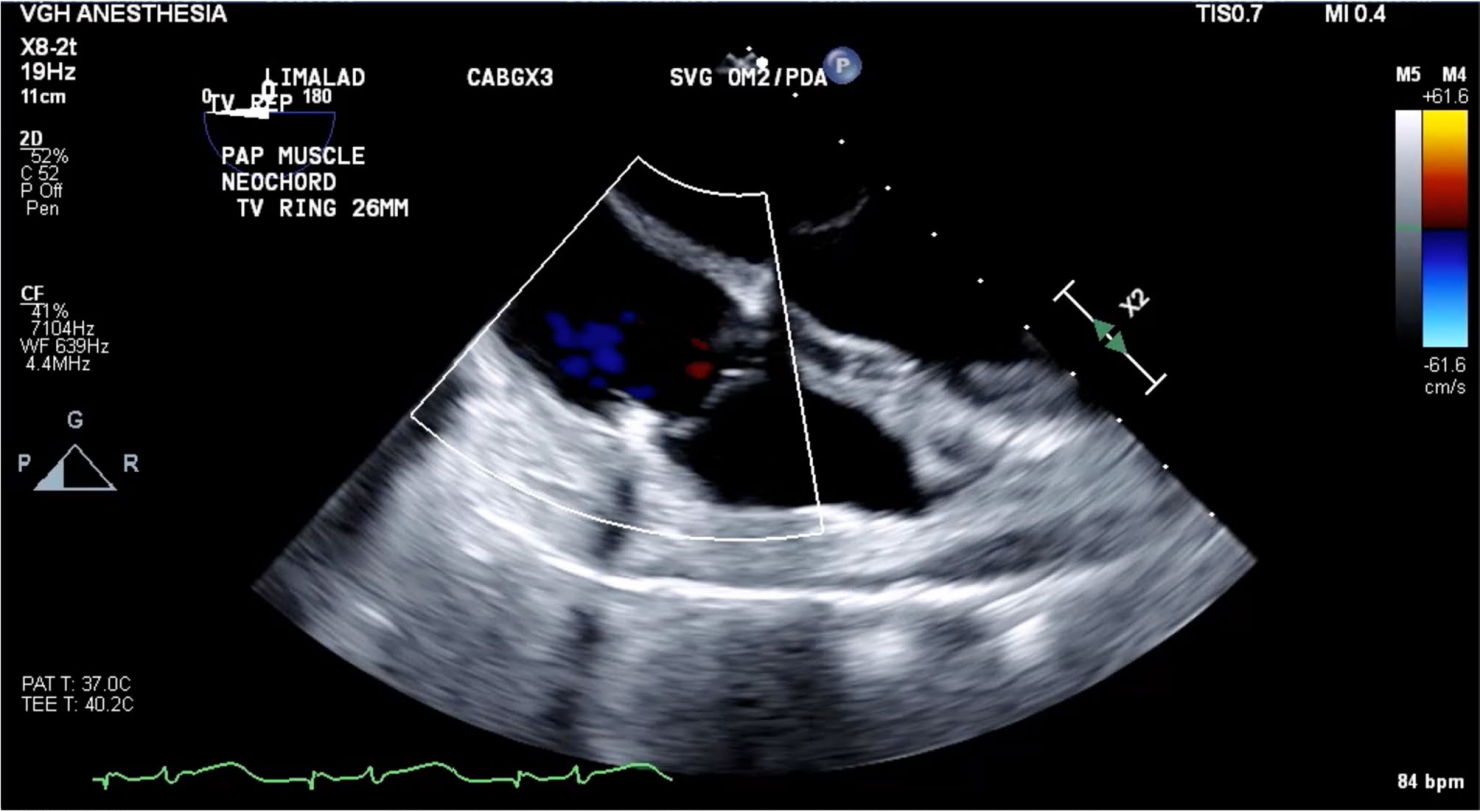
Post tricuspid repair with papillary muscle reinsertion, neochord, and 26 mm ring annuloplasty demonstrating successful repair

The final intraoperative TEE demonstrated mild residual TR with a mean gradient of 1 mmHg and normal biventricular size and systolic function (Fig. 2, Additional File 3). The patient was subsequently weaned off CPB, administered protamine for heparin reversal, and sternotomy closed with no further issues. The patient was transported intubated to the cardiac surgery intensive care unit with low dose milrinone support (0.125mcg/kg/min). Her subsequent postoperative course was uneventful. She was extubated on postoperative day 1 and discharged home from the cardiac surgery ward on postoperative day 5 with no complications.

## Discussion and conclusions

We present a case of a routine isolated CABG patient who developed severe post-CPB TR secondary to a ruptured chordae tendineae. Echocardiographic assessment prior to the institution of CPB revealed only trace TR with normal leaflet motion and morphology. Upon weaning from CPB, however, severe eccentric TR with the appearance of a flail posterior TV leaflet was identified with TEE (Fig. 1). Considering this, the decision was made to investigate the valve surgically given the high suspicion of a structural cause. Visual inspection of the tricuspid valve and subvalvular structure revealed an acutely ruptured chordae tendineae of the posterior leaflet primary. Furthermore, the associated papillary muscle was atypically inserted into the anteroseptal wall, crossing the right ventricle. The differential of structural TR post CPB includes direct trauma, ischemia from poor myocardial protection, ischemia from graft dysfunction, and acute afterload on the right ventricle from pulmonary hypertension. In this case, injury to the subvalvular TV apparatus from artiocaval cannulation is less likely given this was an uneventful cannulation and there was no evidence of new tricuspid regurgitation on TEE after cannulation and prior to the institution of CPB. The venous cannula was also not removed prior to the identification of the new tricuspid regurgitation on TEE. Suboptimal myocardial protection and resultant myocardial is also unlikely given the frequency and dose of cardioplegia. Additionally, the quality of all grafts were confirmed to be adequate with both direct flow measurement after completion of the distal anastomosis and via a biphasic doppler signal during the first attempt at weaning from CPB. There was no acute increases in afterload for the right ventricle and there was no protamine given after the first CPB wean. Given the anomalous papillary muscle location, it was postulated by our group lead by a consultant senior cardiac surgeon with expertise in complex tricuspid valve disease that routine manipulation of the heart during CABG may have played a role in the anomalous papillary muscle chordae tendineae rupture. In the process of obtaining adequate surgical exposure for the PDA anastomosis, the heart is routinely retracted. In this case, this anomalous papillary muscle may have experienced abnormal stress given its atypical anatomical orientation. This tension likely led to the ruptured chordae tendineae and resultant flail posterior TV leaflet. Of note, the TV looked morphologically normal and there was no pulmonary artery catheter inserted at any time during the case, ruling this out as a potential causative mechanism of injury.

The use of intraoperative TEE during cardiac surgery is supported by various societal practice guidelines, the most recent being a joint publication from the American Society of Echocardiography and the Society of Cardiovascular Anesthesiologists in 2010 [4]. These guidelines recommend routine intraoperative TEE in all adults without contraindications undergoing both open heart and thoracic aortic surgical procedures [4]. As a result, the use of intraoperative TEE in these surgical populations has now become a widely accepted standard of practice in North America. However, these guidelines only recommend clinicians consider the use of intraoperative TEE in patients undergoing isolated CABG [4]. As such, the use of intraoperative TEE in the isolated CABG population is subject to significant practice variation.

The use of intraoperative TEE during isolated CABG has increased significantly over recent years. A retrospective cohort study looking at planned CABG procedures from the Society of Thoracic Surgeons Adult Cardiac Surgery Database demonstrated in 2019, 62.1% of isolated CABG procedures utilized intraoperative TEE, up from 39.9% in 2011 [5]. This increase reflects its benefits in improving surgical outcomes, such as identifying occult valvular pathologies that may necessitate additional interventions during surgery. Indeed, the study revealed intraoperative TEE was associated with a higher occurrence of unplanned valve procedures during isolated CABG, as well as lower operative mortality [5].

In this case of a routine CABG necessitating a complex TV repair, intraoperative TEE played a critical role in the immediate identification of this rare, iatrogenic cause of TR. The case highlights how intraoperative TEE can facilitate the early detection and correction of not only existing structural pathologies, but also unexpected structural changes caused by iatrogenic complications, to improve patient outcomes. This premise has been supported by several other studies of unplanned valvular repair performed during routine, isolated CABG [6, 7]. The case we present here is unusual in that it describes a rare iatrogenic complication that has not been previously reported in a patient undergoing CABG. This case lends its support to the routine use of intraoperative TEE in isolated CABG patients to allow for a more comprehensive assessment of structural pathology that extends beyond the primary surgical target.

New or worsening tricuspid regurgitation upon weaning from CPB is often functional in etiology. However, iatrogenic structural causes are possible and warrant thorough investigation with a high index of suspicion. Intraoperative TEE was essential for the prompt diagnosis and surgical correction of a rare anomalous papillary muscle chordae rupture during routine CABG. This case supports the routine use of intraoperative TEE in isolated CABG surgery.

## Supplementary Information

Supplementary Material 1. Video 1.mov. Severe tricuspid regurgitation with evidence of right ventricular dysfunction on mid esophageal view

Supplementary Material 2. Video 2.mov. Flail segment posterior segment of the tricuspid valve on a deep transgastric right ventricular view.

Supplementary Material 3. Video 3.mov. Mild residual tricuspid regurgitation and normal biventricular size and systolic function.

## Data Availability

No datasets were generated or analysed during the current study.

## Abbreviations

TR: Tricuspid Regurgitation
TV: Tricuspid Valve
CPB: Cardiopulmonary Bypass
CABG: Coronary Artery Bypass Grafting
TEE: Transesophageal Echocardiography
CAD: Coronary Artery Disease
CCTA: Coronary Computed Tomography Angiography
LAD: Left Anterior Descending Coronary Artery
LCx: Left Circumflex Coronary Artery
RCA: Right Coronary Artery
ACT: Activated Clotting Time
LIMA: Left Internal Mammary Artery
PDA: Posterior Descending Artery
OM1: First Obtuse Marginal Coronary Artery
